# Supplementary material for: Behavioural activation for depressive symptoms in young people with emerging or early psychosis: A pilot study protocol
Source: PLoS One. 2023 Jan 20;18(1):e0280559. doi: 10.1371/journal.pone.0280559 (PMC9858082; doi:10.1371/journal.pone.0280559)
Supplement: S1 Protocol — (DOCX) [file pone.0280559.s002.docx]

**Behavioural Activation for depressive symptoms in young people with emerging or early psychosis: A pilot study protocol**

Investigators: Prof M Byrne, Prof R Gray, Ms R Creek, Dr M Jones, Dr E Brown, Dr B Nic Giolla Easpaig, Dr D Mitchell, Prof B Tan, Mr S Denis. *Prof D Bressington *Lead Investigator

**Behavioural Activation for depressive symptoms in young people with emerging or early psychosis: A pilot study protocol**

**Introduction**

Psychosis is commonly characterized by the misinterpretation of or difficulties in accurately perceiving reality [1]. The symptoms of psychosis often emerge in young people, where in 80% of cases the first episodes of psychosis occur sometime between the late teenage years and 30 years of age [2]. Early intervention for people experiencing a first episode of psychosis (FEP) is essential to improve functioning, promote recovery, and prevent future episodes [3]. More recently, there has been recognition that providing treatment earlier than during that first episode can be of benefit for individuals who are at ‘ultra high risk’ (UHR) of developing psychosis. This time where sub-threshold symptoms are experienced, is also referred to as “emerging psychosis”, “at-risk-mental-state” or “clinical high-risk” [4].

While treatment for an individual’s FEP often results in remission of psychotic symptoms, co-morbid psychiatric diagnoses are common. One of the most common among young people with emerging or early psychosis (YPEEP) is depressive symptoms occurring in up to 75% of the YPEEP population [5]. Co-occurring depression is associated with poor outcomes, including a 59% increase in suicidal behaviour [6], a higher frequency of hospital admissions [7] and a reduction in occupational and social functioning [8]. Depressive symptoms may also have a role in progressing the psychosis prodrome [9, 10] and improvements in depressive symptoms are associated with reductions in psychotic-type symptoms in young people [11]. There is clear evidence that Cognitive Behavioural Therapy (CBT) can be effective at treating depression in YPEEP [12-14]. However, CBT is a complex/expensive intervention requiring clinicians to undergo specialist training and deliver complex treatment protocols [12]. Hence, it is essential to establish easily accessible, safe and effective psychosocial treatments for depression in YPEEP.

Evidence from meta-analyses has shown Behavioural Activation (BA) therapy to be as effective at treating depressive symptoms as CBT in the general population [15-17]. Furthermore, a systematic review and meta-analysis has reported the promising effect, feasibility, and acceptability of BA specifically in the treatment of depression among young people [18]. Theoretically, BA may also help YPEEP experiencing depression, assisting them to reconnect with positive experiences by using activity monitoring and goal-led activity scheduling [15]. Importantly, BA is an intervention that can be delivered with high levels of fidelity by non-specialist clinicians and requires minimal training [17, 19], which would be of particular benefit in areas with a lack of psychologists and other staff with specialist training.

To the best of our knowledge there has been no previous trial evaluating the acceptability and feasibility of BA interventions for a YPEEP population. This project would be the first to establish the feasibility of clinician delivered BA as an adjunct to standard care to reduce depression in YPEEP. These novel findings will inform the design of a full, appropriately powered randomized controlled trial.

**Study aim and objectives**

The overall aim will be to train at least eight clinicians in BA using an established University accredited online “Professional Certificate in BA for Depression” program and support them to deliver BA to improve depressive symptoms in YPEEP. Specifically, the objectives of the pilot trial are to:

1. Establish the proportion of YPEEP with clinically meaningful depression symptoms.
2. Establish the proportion of clinicians that complete the BA training and are deemed to be competent.
3. Determine the proportion of eligible participants approached who agree to consent to the research.
4. Determine the proportion of participants that complete baseline measures, complete BA treatment (attending for a minimum of six sessions over the six weeks), and complete follow-up measures (immediately post intervention and at 3 months follow-up).

Additionally, we will:

1. Establish clinicians’ fidelity to treatment.
2. Calculate preliminary efficacy of BA on clinical outcome measures.
3. Explore participants’ experiences of facilitating BA (clinicians) and receiving BA (YPEEP).

**Materials and methods**

**Study design**

The feasibility and acceptability of delivering BA to YPEEP clients will be assessed using a pilot controlled clinical trial with a two-arm parallel-group design. This pilot trial protocol is registered with the Australian New Zealand Clinical Trials Registry (ref no. ACTRN12622000756729). The ‘Standard Protocol Items: Recommendations for Interventional Trials’ (SPIRIT) [20, 21] is used to guide the presentation of the current study protocol (see S1 File: Completed SPIRIT checklist). The pilot trial will be conducted in accordance with IHI Good Clinical Practice guidelines. A trial steering committee will be convened to oversee and guide the research as needed.

**Study setting**

This study will be carried out in a youth early psychosis service in Australia between June 2022 and October 2023 known as *headspace* Early Psychosis (hEP) [22]. The early psychosis service is one of the services offered by a youth mental health centre providing mental health services to young people aged 12-25. The early psychosis service had 102 YPEEP clients receiving treatment in the first quarter of 2021 and receive an average of 12 new referrals each month. Additional hEP treatment centres will be recruited where necessary.

**Participants**

*Inclusion criteria for clinicians*

Any clinician with at least 6 months of experience of working with young people with early or emerging psychosis, who is working with clients at the early psychosis service will be eligible to take part. Examples of clinician who may work in these roles include, but are not limited to; registered nurses, social workers, youth peer support workers, occupational therapists, psychologists and psychiatrists.

*Inclusion criteria for YPEEP clients*

Clients aged 15 years and older who are engaged with the early psychosis service will be eligible to participate if they:

1. have experienced early/emerging psychosis (<5 years), received ≥1 month of care, and are experiencing depressive symptoms [BPRS depression item score ≥3, according to the most recent BPRS assessment conducted by clinical staff within routine clinical practice];
2. are able to understand and speak English; and
3. are able to provide informed consent.

Clients will be excluded if they have a primary diagnosis of substance misuse (i.e. that the psychosis or UHR state is secondary to substance misuse disorder), express suicidal ideation or present a known risk to themselves/others.

**Sample size**

There has been some degree of ambiguity about sample size requirements for feasibility studies and there are no previous studies which have evaluated BA in YPEEP which could be used to guide this estimate. Therefore, our sample size estimation will be based on recommendations from the literature that feasibility studies ought to aim for sample sizes of between 24 and 50 participants [23, 24]. Furthermore, a systematic review of UK registered feasibility studies reported the median sample size with continuous outcome measures to be 30 participants per group [25]. Based on this guidance, our target sample size is 60 participants (30 in each group). This estimate will facilitate the generation of information about sample size requirements for a subsequent full-scale trial and is not intended to be sufficiently powered to detect statistically significant differences in outcomes between groups.

**Recruitment**

An information sheet about the research will be provided to and discussed with eligible clinicians who are staff members working in the early psychosis service. For the clinicians who are willing to participate, they will be asked to review the informed consent form and confirm their agreement to participate by signing this form. Potential YPEEP participants will be initially identified by clinicians, who will determine whether the young person meets the study inclusion criteria.

Potential YPEEP participants will be briefly informed about the study by their clinician and offered a study flyer containing more detailed information. If the young person is interested in taking part, they will be asked for their permission to be approached by a trained research assistant. If permission is granted, the research assistant will then provide each potential participant with a detailed study information sheet. The research assistant can offer further explanation or clarification and address any questions or concerns potential participants may have, as needed. Potential participants will be encouraged to discuss the study with their families and other appropriate people and will be given adequate time (at least 48 hours) to consider the invitation. Eligible young people who are 18 years of age and older and wish to participate, will be asked to review the informed consent form and confirm their agreement to participate by signing this form. If the eligible young person wishing to participate is less than 18 years of age, consent will be sought from their parent/guardian, in addition to obtaining the informed consent of the young person. Recruitment will cease when the planned sample size is reached.

At the conclusion of the intervention, the researchers will invite a subsample of clinicians and YPEEP participants to take part in a semi-structured interview. Clinicians and YPEEP participants will be advised on their respective information sheets and informed consent forms that they can participate in the trial and later decide not to participate in an interview. A purposive sample of a minimum 4 clinicians and 12 YPEEP participants will be interviewed 3-months post intervention (additional interviews may be conducted until data saturation is met). YPEEP participants will be purposively sampled to capture the range and diversity of experiences; based on their attendance at the BA sessions, engagement with the trial more generally and clinical outcomes. A member of the research team will contact selected clinicians and YPEEP participants and invite them to be interviewed. Consent to participate in the interview will be reconfirmed prior to arranging the interview and potential interviewees will be reminded of their rights in the study.

**Random treatment allocation and blinding**

Baseline data will be collected from YPEEP participants who will then be randomly assigned into either: 1) the BA group, who will receive BA therapy in addition to TAU or 2) the TAU group, who will receive the standard care currently offered by the service. Random treatment allocation will be generated by an external computerized randomization service which uses block randomization with random permuted block sizes to ensure appropriate allocation concealment and equal samples across groups. The external randomisation service will hold the allocation sequence and inform the trial coordinator of participants’ group allocation via text message once baseline data has been collected from consenting participants. The trial coordinator then will inform the participant and clinician of treatment allocation. Allocation will be blinded to the statistician, but it will not be masked to the clinicians and research assistants who will collect baseline, post-intervention, and follow-up outcome data.

**Intervention**

*BA Intervention group*

Participants in the intervention group will receive BA treatment in addition to standard care. These participants will be offered 12 sessions of BA, which will be delivered twice weekly, in 30-minute sessions, for six weeks. Participants will be deemed to have received an adequate ‘dose’ of the intervention if they have attended 6 BA sessions over the 6-week period (i.e. 50%). The BA treatment will follow a treatment protocol based on previous trials of BA for depression [26], the COBRA trial protocol [27] and the National Institute for Health and Care Excellence recommendations for the frequency and duration of BA therapy [28]. Specific BA techniques that will be applied include the: identification of depressed behaviours; analysis of the triggers and consequences of depressed behaviours; monitoring of activities; development of alternative goal-orientated behaviours; scheduling of activities; and the development of alternative behavioural responses to rumination.

*TAU group*

Every participant in the control group will receive routine standard early intervention in psychosis services and no other specific intervention will be provided. Across sites, TAU is composed of case management and antipsychotic medication. Additionally, TAU may involve family support, psychosocial groups, vocational support, peer support as well as care for physical health. Multidisciplinary teams responsible for delivering standard care services usually consist of psychologists, psychiatrists, mental health nurses, occupational therapists, and social workers. The TAU condition will ensure appropriate, ongoing quality care for this group of young people. Young people at UHR status should receive care for at least 12 months and those with FEP for two years [22].

*BA training*

Eight clinicians will be recruited and trained to deliver the intervention. Each clinician will deliver the intervention to approximately five YPEEP participants. The clinicians will be trained in BA techniques using an academically accredited and established online “Professional Certificate in BA for Depression” program offered by the University of South Australia (facilitated by MJ and SD). The 10-week online training consists of five modules (the evidence base of BA; introduction to BA; assessment and mood monitoring; functional analysis; and activity scheduling).

The expected learning outcomes upon completion of the online training program are that the clinicians will acquire a sound understanding of the BA intervention, develop the relevant core skills, and demonstrate competency in BA delivery. Trainees are assessed using multiple choice questionnaires at the end of each module and via an assessment of an audio recorded Behavioural Activation Assessment and an Activity Schedule with a work colleague. Following the satisfactory completion of the training, clinicians will be supported through monthly, online clinical supervision sessions provided by the researchers who are experienced in teaching and supervising BA trainees.

*Intervention fidelity*

Intervention fidelity will be established via recording randomly selected treatment sessions and completing a fidelity checklist. Two sessions of BA treatment will be randomly chosen from each clinician and audio-recorded (with the clinician’s and participants’ prior consent) by the researchers to monitor fidelity to the treatment protocol. The fidelity checklist [29] will be used by two researchers, independently, to assess the clinician’s adherence to BA treatment protocol. The schedule of enrolment, intervention and assessments is shown in Figure 1.

Fig 1. Schedule of enrolment, interventions, and assessments


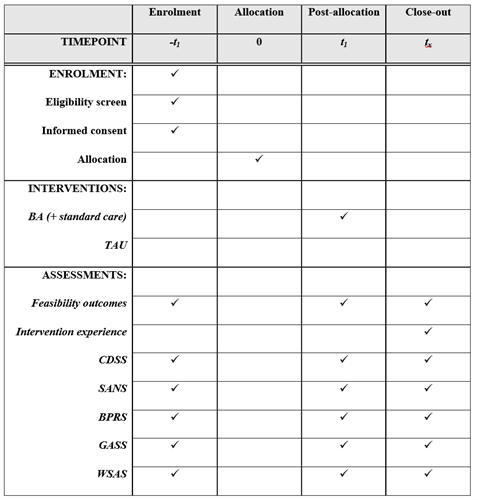


**Evaluation**

*Feasibility*

The study feasibility outcomes will be evaluated by calculating a) the proportion of YPEEP with clinically meaningful depression symptoms; b) the proportion of clinicians that complete the BA training and the proportion deemed to be competent; c) the proportion of eligible participants approached who agree to consent to the research; d) the proportion of YPEEP participants that complete baseline measures, complete BA treatment (attending for at least six sessions), and complete follow-up measures (immediately post intervention and at 3 months follow-up).

*Experiences of the intervention/trial*

Semi-structured interviews will be undertaken with a subset of clinicians (minimum of 4) and YPEEP participants (minimum of 12) to explore their experiences of involvement in the pilot trial. Interviews will be conducted with clinicians to gain insight into their experiences of delivering BA therapy and the BA training/supervision. Interviews with YPEEP participants will focus on their experiences of engaging in the BA program and participating in the study. General topic areas (for YPEEP and clinicians) will include: what worked; what did not work; what could be done differently; what could be improved; and what were the facilitators and barriers to engaging with/facilitating the intervention. The young person will be offered the option of having a parent/guardian or support person present in the interview, if desired. The interviews will be conducted by an experienced researcher (in-person or by phone) and are anticipated to last no more than 30 minutes. Data saturation will be determined in relation to the range and diversity of experiences; based on attendance at the BA sessions, engagement with the trial more generally and clinical outcomes. Additional interviews may be conducted so to achieve data saturation.

**Preliminary intervention effects (primary and secondary outcomes)**

The primary outcome will be the assessment of depressive symptoms. The secondary outcome will be the assessment of negative symptoms, overall psychiatric symptoms, medication side effects, and functioning. Outcome measures will be completed at baseline (0 weeks), immediately post-intervention (6 weeks) and 3-months post-intervention.

Depressive symptoms: The depressive symptoms will be measured with Calgary Depression Scale for Schizophrenia (CDSS) [30]. The CDSS is a 4-point Likert type scale (0, absent; 1, mild; 2, moderate; 3, severe) with nine items. It is considered to be a reliable scale, with high internal consistency (Cronbach's alpha= 0.855), and the CDSS is suitable for evaluating symptoms of depression in people at clinical high risk for psychosis [31], including young people [32].

Negative symptoms: The Scale for Assessment of Negative Symptoms (SANS) [33] will be used to assess the negative symptoms of schizophrenia. SANS is 6-point Likert type scale (from 0, none to 5, severe), comprised of five scales each of which assess aspects of negative symptoms; alogia, affective blunting, avolition-apathy, anhedonia-asociality, and attentional impairment. The SANS has demonstrated a high level of reliability, with an overall Cronbach’s alpha ranging from 0.89 to 0.95 and global intraclass correlation coefficient (test-retest reliability) of more than 0.82 in different studies [33-35].

Psychiatric symptoms: The Brief Psychiatric Rating Scale (BPRS) [36] is a 7-point Likert type scale (1, not present-7, extremely severe) which is used to rate psychiatric symptoms such as anxiety, depression, and psychoses. The BPRS has demonstrated good reliability, with overall Cronbach’s alpha value of 0.69 [37] and an overall intra-class correlation of R = 0.78 (p< 0.001) [38].

Medication side effects: The Glasgow Antipsychotic Side-effect Scale (GASS) [39] is 4-point Likert type scale (from 0 point, never to 3 point, everyday) containing 22 items. It is a self-report rating scale which measures an individual’s viewpoint about side effects of antipsychotic medication. The GASS has demonstrated a good test-retest reliability (kappa value=0.72) [39] and internal consistency reliability with Cronbach's alpha ranging from 0.793 to 0.903 [40-42].

Functioning: The Work and Social Adjustment Scale (WSAS) [43] will be used to assess the impact of early/emerging psychosis on various aspects of functioning. The WSAS is a five item self-reported, nine-point Likert scale. It measures how illness impacts on (1) ability to work, (2) home management, (3) social leisure, (4) private leisure and (5) ability to form and maintain close relationships. The Cronbach’s alpha ranges from 0.70 to 0.94 and the overall test–retest correlation score is 0.73 [44].

**Data collection and management**

The trained research assistant will facilitate the data collection process. With prior written consent, participant demographic information will be collected through the early psychosis service at the study site. For the primary (depressive symptoms) and secondary outcomes (negative symptoms, overall psychiatric symptoms, medication side effects, functioning), the data will be collected at baseline, 6 weeks post-intervention and 3 months post-intervention respectively. There are no plans for specific activities to promote participant retention other than the BA intervention being facilitated by participants’ regular clinician and by keeping participant burden to a minimum. With participants’ permission, all outcome data will be collected and analysed from participants who discontinue the study or deviate from the intervention protocol.

The qualitative interviews will be audio-recorded and will subsequently be transcribed. The researcher conducting the interview will ensure that there is no patient identifiable information (e.g. names, addresses) on the audio recording. A professional transcription service with appropriate confidentiality and data security policies will be engaged to transcribe digital recordings of the interviews. Transcriptions will be de-identified by the research team to ensure anonymity. Subsequently, audio-recordings will be securely destroyed. Data will be stored securely on the university’s server and files will be password protected. Only the relevant members of the research team will have access to these files. In accordance with NHMRC guidelines for clinical trials, data will be retained for 15 years and then securely destroyed. Data will be managed in accordance with the National Statement on Ethical Conduct in Human Research and the Charles Darwin University Research Data Management Procedures [45].

**Data analysis**

*Qualitative data analysis*

Qualitative interview data will be content analysed from a phenomenological perspective following Braun and Clarke’s thematic analysis procedure [46]. The planned analysis encompasses the processes of immersion in the data set, line by line coding so to generate a set of preliminary codes, the development of tentative themes, reflection upon and revision of themes as needed before defining and describing finalised themes. The analysis will be inductively-oriented and data-driven. Codes and themes will be validated by an independent researcher at key points in the analysis. NVivo Pro software version 12 will be employed to facilitate the analysis.

*Statistical analyses*

Raw data will be entered and analysed with SPSS (the Statistical Package for the Social Sciences) for Windows, version 26. The demographic characteristics and baseline data measure (mean scores) of all participants will be compared between the two study groups, using Chi-squared test or independent samples t-test to assess the homogeneity of the two groups. The generalized estimating equation (GEE) will be employed to analyse the preliminary effects of BA across the three points (baseline, and 6-weeks and 3-months after intervention) following the intention-to-treat principle, and relevant effect sizes will be calculated. The GEE analysis will account for intra-correlated repeated outcome data and accommodate data missing at random. Feasibility data will be summarized with descriptive statistics. T-tests will be carried out to examine any significant statistical differences between the two arms on primary and secondary outcome measures at both follow-up measurements if the statistical significance of the overall treatment effect was found in the GEE analysis. We will also conduct Chi-squared test or independent samples t-tests (depending on normality of data) to assess significant differences in baseline data measures between participants that discontinue the study and those that adhere to the study protocol/com. In all tests, the level of significance will be set at P values of < 0.05.

**Dissemination**

The Consolidated Standards of Reporting Trials (CONSORT) framework [47] (Figure 2.) will be followed when reporting the present pilot trial findings.

Fig 2. CONSORT framework for reporting


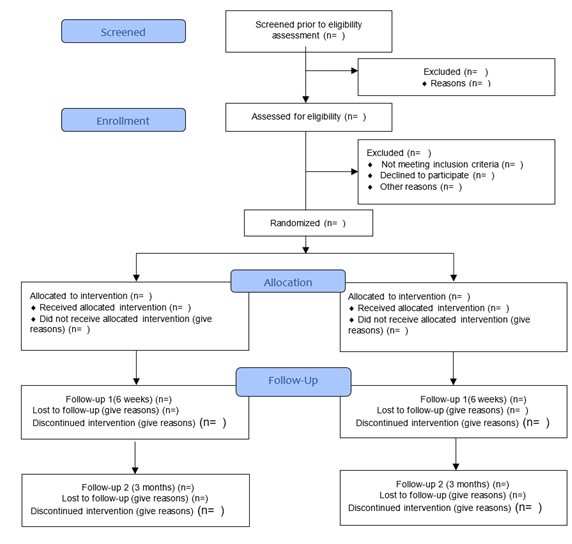


The findings of the study will be written up and submitted for publication. Authorship of any publications will be determined using the recommendations by International Committee of Medical Journal Editors concerning authorship. Findings will also be presented at relevant scholarly and professional conferences. Additionally, the participating study sites will be informed of the report so that they may distribute copies of the report to participants, if desired. Public access to the study protocol will be ensured via the publication of the protocol in a professional peer-reviewed journal and registration of the trial protocol in a publicly accessible database.

**Ethical approval**

Ethical approval for the study has been granted by the Charles Darwin University Human Research Ethics Committee (ref no. H22003) and the appropriate ethical approval/governance approvals will be obtained from the early psychosis service at the study site. Potential participants will be aware that their participation is entirely voluntary and that they may withdraw from the study at any point without penalty. Participants aged <18 years will also need to provide parental/guardian consent to take part in the study. Recruitment will be conducted by a trained research assistant who is not working at the service provider in order to minimise the risk that participants may feel coerced to take part (please see “Recruitment” section for more detailed information).

The intervention will be delivered by the YPEEP participant’s regular clinician who will be responsible for recognizing, responding and reporting any distress arising from the intervention (i.e. debriefing). After each YPEEP participant has completed each session of BA, the clinician will ask them about their emotional well-being and if they have experienced any distress. If distress is experienced, the participants will have access to ongoing support through their existing clinical/support team and will be aware of how to contact this team. Approval will be sought for any modifications to the protocol from the ethics committees noted above and amendments will be made to the documentation submitted to the Australian New Zealand Clinical Trials Registry, accordingly.

**Patient and public involvement**

A youth advisory committee was consulted to develop and refine the study recruitment materials. The committee provided input to help ensure that the texts were clear and in the best possible format for young people who are attending the service to engage with. All of the suggestions from the committee were implemented. An expert by experience consultant who has lived experiences of psychosis will join the trial steering committee, in order to facilitate consumer input and guidance in this forum.

**Safety considerations**

As noted, BA has not been conducted in YPEEP before. Thus, safety considerations are formulated on the basis of studies conducting similar interventions and/or those using psychosocial interventions with the same patient population. An RCT study investigating cost-effectiveness of BA compared with CBT for adults with depression reported that depression-related, but not treatment-related, serious adverse events occurred in three participants in the BA group and eight participants in the CBT group [27]. We considered that low intensity CBT for psychosis is very similar to BA as it adopts behavioural interventions in conjunction with graded exposure [48]. A systematic review of low intensity CBT for psychosis in adults including 10 studies reported that that the intervention seemed safe (no mention of adverse events) and shows promise for psychotic symptoms [49]. The individual study most similar to our study is a pilot study of low intensity CBT for psychosis, in which the main elements of the intervention included BA and graded exposure [48]. The small study reported that staff and participant satisfaction was high, with no adverse events reported. Therefore, based on the best available evidence, a reasonable conclusion is that BA for young people with early psychosis is likely to be broadly safe and acceptable.

In order to mitigate the risk of adverse effects we will put in place several procedures. We will ensure that researchers and clinicians engage with participants at each assessment or at the start of each BA session to check how they are feeling and if they experienced any unexpected effects of the intervention. The BA will be also be delivered by participants’ routine community-based clinician, thus if the participants show any signs of suicide/self-harm risk they will be able to activate their existing risk management plan to ensure the person is supported and referred on for additional treatment as required. In addition, we will apply Good Clinical Practice best practice advice about recording and coding adverse events. All adverse events will be reported to the trial steering committee. If the trial steering committee considered that adverse events are directly associated with the intervention, we will halt the trial as appropriate.

**Discussion**

While findings from systematic reviews and meta-analyses would suggest an important role for BA in the treatment of depression [15, 18], evidence is needed about the acceptability and feasibility of this intervention for YPEEP. This pilot trial will be undertaken in partnership with a youth early psychosis service, wherein clinicians will be trained and supported in the delivery of this therapeutic intervention. As far as we are aware, this trial will be the first to establish the acceptability and feasibility of clinician-delivered BA for this population, and as such, will provide a vital foundation for the design of a full-scale randomised controlled trial. To date, the study has received ethical approval and the protocol has been registered with the Australian New Zealand Clinical Trials Registry. Recruitment will commence in July 2022. It is anticipated that data collection will be completed by December 2022. Subsequently analysis and writing up of the findings will be undertaken between December 2022 and February 2023.

In considering potential risks, it is possible that the target sample size is not reached within the anticipated timeframe, and the project would be delayed. Further, we may need to make some variations to ensure adherence with local and national COVID-19 public health measures, which could include the way in which care is delivered in the service and/ or how the study is administered. A limitation of the research is that the qualitative findings cannot be generalised, and instead, ought to be interpreted relative to the relevant study context. Nevertheless, given the potential efficacy of BA, in addition to its minimal implementation burden within services [17, 19], this research will generate valuable evidence for the range of organisations involved in treating and supporting YPEEP.

**References**

1. Early Psychosis Guidelines Writing Group and EPPIC National Support Program. Australian Clinical Guidelines for Early Psychosis [Internet]. Melbourne, Australia: Orygen, The National Centre of Excellence in Youth Mental Health, 2016 [cited 2022, Jun]. Available from: https://www.orygen.org.au/Campus/Expert-Network/Resources/Free/Clinical-Practice/Australian-Clinical-Guidelines-for-Early-Psychosis/Australian-Clinical-Guidelines-for-Early-Psychosis.aspx?ext

2. Chee GL, Wynaden D, Heslop K. The physical health of young people experiencing first-episode psychosis: Mental health consumers' experiences. Int J Ment Health Nurs. 2019;28(1):330-8. doi: 10.1111/inm.12538.

3. Galletly C, Castle D, Dark F, Humberstone V, Jablensky A, Killackey E, et al. Royal Australian and New Zealand College of Psychiatrists clinical practice guidelines for the management of schizophrenia and related disorders. Aust N Z J Psychiatry. 2016;50(5):410-72. doi: 10.1177/0004867416641195.

4. Catalan A, Salazar de Pablo G, Vaquerizo Serrano J, Mosillo P, Baldwin H, Fernández-Rivas A, et al. Annual research review: prevention of psychosis in adolescents – systematic review and meta-analysis of advances in detection, prognosis and intervention. J Child Psychol Psychiatry. 2021;62(5):657-73. doi:10.1111/jcpp.13322.

5. Upthegrove R. Depression in schizophrenia and early psychosis: implications for assessment and treatment. Adv Psychiatr Treat. 2009;15(5):372-9. doi: 10.1192/apt.bp.108.005629.

6. McGinty J, Sayeed Haque M, Upthegrove R. Depression during first episode psychosis and subsequent suicide risk: a systematic review and meta-analysis of longitudinal studies. Schizophr Res. 2018;195:58-66. doi: 10.1016/j.schres.2017.09.040.

7. an der Heiden W, Könnecke R, Maurer K, Ropeter D, Häfner H. Depression in the long-term course of schizophrenia. Eur Arch Psychiatry Clin Neurosci. 2005;255(3):174-84. doi: 10.1007/s00406-005-0585-7.

8. Sands JR, Harrow M. Depression during the longitudinal course of schizophrenia. Schizophr Bull. 1999;25(1):157-71. doi: 10.1093/oxfordjournals.schbul.a033362.

9. Häfner H, an der Heiden W, Maurer K. Evidence for separate diseases? Eur Arch Psychiatry Clin Neurosci. 2008;258(2):85. doi: 10.1007/s00406-008-2011-4.

10. Myles-Worsley M, Weaver S, Blailes F. Comorbid depressive symptoms in the developmental course of adolescent-onset psychosis. Early Interv Psychiatry. 2007;1(21):183-90. doi: 10.1111/j.1751-7893.2007.00022.x.

11. Yung AR, Buckby JA, Cosgrave EM, Killackey EJ, Baker K, Cotton SM, et al. Association between psychotic experiences and depression in a clinical sample over 6 months. Schizophr Res. 2007;91(1-3):246-53. doi: 10.1016/j.schres.2006.11.026.

12. Addington J, Epstein I, Liu L, French P, Boydell KM, Zipursky RB. A randomized controlled trial of cognitive behavioral therapy for individuals at clinical high risk of psychosis. Schizophr Res. 2011;125(1):54-61. doi: 10.1016/j.schres.2010.10.015.

13. Morrison AP, French P, Stewart SLK, Birchwood M, Fowler D, Gumley AI, et al. Early detection and intervention evaluation for people at risk of psychosis: multisite randomised controlled trial. BMJ. 2012;344:e2233. doi: 10.1136/bmj.e2233.

14. Singer, AR, Addington, DE, Dobson, KS, Wright C. A pilot study of cognitive behavior therapy for depression in early psychosis. Cogn Behav Pract. 2014; 21(3):323-334. doi:10.1016/j.cbpra.2013.08.004.

15. Welsh P, Kitchen CEW, Ekers D, Webster L, Tiffin PA. Behavioural activation therapy for adolescents ‘at risk’ for psychosis? Early Interv Psychiatry. 2016;10(2):186-8. doi:10.1111/eip.12155.

16. Mazzucchelli T, Kane R, Rees C. Behavioral activation treatments for depression in adults: a meta-analysis and review. Clin Psychol. 2009;16(4):383-411. doi:10.1111/j.1468-2850.2009.01178.x.

17. Uphoff E, Ekers D, Robertson L, Dawson S, Sanger E, South E, et al. Behavioural activation therapy for depression in adults. Cochrane Database Syst Rev. 2020;(7). doi:10.1002/14651858.CD013305.pub2.

18. Martin F, Oliver T. Behavioral activation for children and adolescents: a systematic review of progress and promise. Eur Child Adolesc Psychiatry. 2019;28(4):427-41. doi: 10.1007/s00787-018-1126-z.

19. Cuijpers P, van Straten A, Warmerdam L. Behavioral activation treatments of depression: a meta-analysis. Clin Psychol Rev. 2007;27(3):318-26. doi: 10.1016/j.cpr.2006.11.001.

20. Chan A, Tetzlaff JM, Altman DG, Laupacis, A, Gøtzsche, PC, Krleža-Jerić, K et al; SPIRIT 2013 statement: defining standard protocol items for clinical trials. Ann Intern Med. 2013;158:200-207. doi:10.7326/0003-4819-158-3-201302050-00583

21. Chan A, Tetzlaff JM, Gøtzsche, PC, Altman DG, Mann H, Berlin J A et al. SPIRIT 2013 explanation and elaboration: guidance for protocols of clinical trials BMJ 2013; 346 :e7586 doi:10.1136/bmj.e7586

22. Brown E, Gao CX, Staveley H, Williams G, Farrelly S, Rickwood D, et al. (2021). The clinical and functional outcomes of a large naturalistic cohort of young people accessing national early psychosis services. Aust N Z J Psychiatry. 2021;30. doi: 10.1177/00048674211061285

23. Sim J, Lewis M. The size of a pilot study for a clinical trial should be calculated in relation to considerations of precision and efficiency. J Clin Epidemiol. 2012;65(3):301-8. doi:10.1016/j.jclinepi.2011.07.011.

24. Julious SA. Sample size of 12 per group rule of thumb for a pilot study. Pharm Stat. 2005;4(4):287-91. doi: 10.1002/pst.185.

25. Billingham SA, Whitehead AL, Julious SA. An audit of sample sizes for pilot and feasibility trials being undertaken in the United Kingdom registered in the United Kingdom Clinical Research Network database. BMC Med Res Methodol. 2013;13:104. doi: 10.1186/1471-2288-13-104.

26. Ekers DM, Dawson MS, Bailey E. Dissemination of behavioural activation for depression to mental health nurses: training evaluation and benchmarked clinical outcomes. Int J Ment Health Nurs. 2013;20(2):186-92. doi: 10.1111/j.1365-2850.2012.01906.x.

27. Richards DA, Ekers D, McMillan D, Taylor RS, Byford S, Warren FC, et al. Cost and Outcome of Behavioural Activation versus Cognitive Behavioural Therapy for Depression (COBRA): a randomised, controlled, non-inferiority trial. Lancet. 2016;388(10047):871-80. doi: 10.1016/s0140-6736(16)31140-0.

28. National Institute for Health Care and Excellence. Depression in adults: recognition and management. Clinical guideline [CG90]Published: 28 October 2009. Available from: https://www.nice.org.uk/guidance/cg90/chapter/Recommendations#step-2-recognised-depression-persistent-subthreshold-depressive-symptoms-or-mild-to-moderate

29. Bellg AJ, Borrelli B, Resnick B, Hecht J, Minicucci DS, Ory M, et al. Enhancing treatment fidelity in health behavior change studies: best practices and recommendations from the NIH Behavior Change Consortium. Health Psychol. 2004;23(5):443-51. doi: 10.1037/0278-6133.23.5.443.

30. Addington D, Addington J, Maticka-Tyndale E. Assessing depression in schizophrenia: the Calgary Depression Scale. Br J Psychiatry Suppl. 1993;(22):39-44.

31. Rekhi G, Ng WY, Lee J. Clinical utility of the Calgary Depression Scale for Schizophrenia in individuals at ultra-high risk of psychosis. Schizophr Res. 2018;193:423-7. doi:10.1016/j.schres.2017.06.056.

32. Addington J, Shah H, Liu L, Addington D. Reliability and validity of the Calgary Depression Scale for Schizophrenia (CDSS) in youth at clinical high risk for psychosis. Schizophr Res. 2014;153(1-3):64-7. doi: 10.1016/j.schres.2013.12.014.

33. Andreasen, NC. Scale for the Assessment of Negative Symptoms (SANS). Br J Psychiatry.1989;155(Suppl 7), 53–58.

34. Phillips MR, Xiong W, Wang RW, Gao YH, Wang XQ, Zhang NP. Reliability and validity of the Chinese versions of the Scales for Assessment of Positive and Negative Symptoms. Acta Psychiatr Scand. 1991;84(4):364-70.

35. Charernboon T. Preliminary study of the Thai-version of the Scale for the Assessment of Positive Symptoms (SAPS-Thai): content validity, known-group validity, and internal consistency reliability. Rev Psiquiatr Clín. 2019;46(1):5-8. doi: 10.1590/0101-60830000000183.

36. Overall JE, Gorham DR. The Brief Psychiatric Rating Scale (BPRS): recent developments in ascertainment and scaling. Psychopharmacol Bull. 1988;24(1):97-9.

37. Overall JE, Gorham DR. The Brief Psychiatric Rating Scale. Psychol Rep. 1962;10(3):799-812. doi: 10.2466/pr0.1962.10.3.799.

38. Andersen J, Larsen JK, Kørner A, Nielsen BM, Schultz V, Behnke K, et al. The Brief Psychiatric Rating Scale: schizophrenia, reliability and validity studies. Nordisk Psykiatrisk Tidsskrift. 1986;40(2):135-8. doi: 10.3109/08039488609096456.

39. Waddell L, Taylor M. A new self-rating scale for detecting atypical or second-generation antipsychotic side effects. J Psychopharmacol. 2008;22(3):238-43. doi: 10.1177/0269881107087976.

40. Ignjatović Ristić D, Cohen D, Obradović A, Nikić-Đuričić K, Drašković M, Hinić D. The Glasgow antipsychotic side-effects scale for clozapine in inpatients and outpatients with schizophrenia or schizoaffective disorder. Nord J Psychiatry. 2018;72(2):124-9. doi: 10.1080/08039488.2017.1400097.

41. AlRuthia Y, Alkofide H, Alosaimi FD, Alkadi H, Alnasser A, Aldahash A, et al. Translation and cultural adaptation of Glasgow Antipsychotic Side-effects Scale (GASS) in Arabic. PLoS One. 2018;13(8):e0201225. doi: 10.1371/journal.pone.0201225.

42. Hynes C, Keating D, McWilliams S, Madigan K, Kinsella A, Maidment I, et al. Glasgow Antipsychotic Side-effects Scale for Clozapine - development and validation of a clozapine-specific side-effects scale. Schizophr Res. 2015;168(1-2):505-13. doi: 10.1016/j.schres.2015.07.052.

43. Marks IM. Behavioural psychotherapy: maudsley pocket book of clinical management. Bristol, England: Wright/IOP Publishing; 1986.

44. Mundt JC, Marks IM, Shear MK, Greist JH. The Work and Social Adjustment Scale: a simple measure of impairment in functioning. Br J Psychiatry. 2002;180:461-4. doi: 10.1192/bjp.180.5.461.

45. National Health and Medical Research Council. National statement on ethical conduct in human research 2007 (updated 2018). Canberra, Australia: National Health and Medical Research Council; 2007, 2018.

46. Braun V, Clarke V. Using thematic analysis in psychology. Qual Res Psychol. 2006;3(2):77-101. doi: 10.1191/1478088706qp063oa.

47. Eldridge SM, Chan CL, Campbell MJ, Bond CM, Hopewell S, Thabane L, et al. CONSORT 2010 statement: extension to randomised pilot and feasibility trials. BMJ. 2016;355:i5239. doi: 10.1136/bmj.i5239.

48. Waller H, Garety PA, Jolley S, Fornells-Ambrojo M, Kuipers E, Onwumere J, et al. Low intensity cognitive behavioural therapy for psychosis: a pilot study. J Behav Ther Exp Psychiatry. 2013;44(1):98-104. doi: 10.1016/j.jbtep.2012.07.013.

49. Hazell CM, Hayward M, Cavanagh K, Strauss C. A systematic review and meta-analysis of low intensity CBT for psychosis. Clin Psychol Rev. 2016;45:183-92. doi: 10.1016/j.cpr.2016.03.004.
